# Association between Disease-Specific Quality of Life and Complementary Medicine Use in Patients with Rhinitis in Taiwan: A Cross-Sectional Survey Study

**DOI:** 10.1155/2014/958524

**Published:** 2014-10-28

**Authors:** Malcolm Koo, Kai-Li Liang, Rong-San Jiang, Hsin Tsao, Yueh-Chiao Yeh

**Affiliations:** ^1^Department of Medical Research, Dalin Tzu Chi Hospital, Buddhist Tzu Chi Medical Foundation, No. 2 Minsheng Road, Dalin, Chiayi 62247, Taiwan; ^2^Dalla Lana School of Public Health, University of Toronto, No. 155, College Street, Toronto, ON, Canada M5T 3M7; ^3^Department of Otolaryngology, Taichung Veterans General Hospital, No. 1650, Sec. 4, Taiwan Boulevard, Taichung City 40705, Taiwan; ^4^School of Medicine, Chung Shan Medical University, No. 110, Sec. 1, Jianguo North Road, Taichung City 40201, Taiwan; ^5^Department of Medicine, National Yang-Ming Medical University, No. 155, Sec. 2, Linong Street, Taipei 11221, Taiwan; ^6^Graduate Institute of Natural Healing Sciences, Nanhua University, No. 55, Sec. 1, Nanhua Road, Dalin, Chiayi 62249, Taiwan; ^7^Department of Natural Biotechnology, Nanhua University, No. 55, Sec. 1, Nanhua Road, Dalin, Chiayi 62249, Taiwan

## Abstract

Rhinitis is a common medical condition and can seriously impact patients' quality of life. The objective of this study was to investigate the association between disease-specific quality of life and use of complementary and alternative medicine (CAM) modalities among Taiwanese rhinitis patients. A cross-sectional survey was undertaken at the outpatient department of otolaryngology in a medical center in Taiwan. Sociodemographic information, disease-specific quality of life (Chinese version of the 31-item Rhinosinusitis Outcome Measure, CRSOM-31), and previous use of CAM modalities for treatment of rhinitis of the patients were ascertained. Factor analysis was performed to reduce the number of CAM modalities. The resulting factors were analyzed for their association with CRSOM-31 score using linear regression analyses. Results from the multiple linear regression analyses indicated that Factor 1 (traditional Chinese medicine), Factor 2 (mind-body modalities), Factor 3 (manipulative-based modalities), female sex, and smoking were significantly associated with a worse disease-specific quality of life. In conclusion, various CAM modalities, female sex, and smoking were independent predictors of a worse disease-specific quality of life in Taiwanese patients with rhinitis.

## 1. Introduction

Rhinitis is inflammation of the nasal mucous membranes that can lead to symptoms such as sneezing, nasal congestion, nasal itching, postnasal drainage, and rhinorrhea. Rhinitis is a common medical condition that affects 20 to 40 million people in the United States annually [1]. Although rhinitis is not a life-threatening condition, morbidity from the condition can seriously impair quality of life and increase healthcare cost [2]. It has been suggested that patients with chronic rhinosinusitis had a health status similar to that of patients with arthritis, cancer, asthma, and inflammatory bowel disease. In addition, chronic rhinosinusitis was found to be significantly associated with increased depression and visits to mental-health professionals [3].

Conventional treatment options for management of rhinitis include environmental control, pharmacotherapy, immunotherapy, surgical interventions, and nasal irrigation [4]. Complementary or alternative medicine (CAM) is also commonly used for the treatment of rhinitis [5] and it includes traditional Chinese medicine (TCM) [6], acupuncture [7], herbal remedy [8, 9], probiotics [10], and homeopathy [11]. A population-based survey conducted in Northern California on 300 adults with self-report of a physician diagnosis of rhinosinusitis or asthma found that 42% had used some form of complementary therapies [12]. Another survey on 100 patients attending a rhinology outpatient clinic in Australia reported that 65% of patients had ever used CAM [13].

Previous research has investigated the association between disease-specific quality of life and CAM use among patients with various diseases. A systematic review of the effects of CAM interventions, including yoga, meditation or mindfulness, energy healing, medical qigong, homoeopathy, or mistletoe therapy, on the quality of life in cancer survivors had mixed results [14]. Another meta-analysis of the association between oral Chinese herbal medicine and quality of life in patients with stable chronic obstructive pulmonary disease showed promising results. Nevertheless, the presence of methodological deficiencies, particularly the lack of double-blind method, required cautious interpretation of the study conclusions [15]. In another systematic review of quality of life among patients suffering from pain associated with the spine, acupuncture was found to improve physical functioning but offered only a delayed and small effect on mental functioning [16].

To our knowledge, no studies have specifically evaluated the association between disease-specific quality of life and CAM use among patients with clinically verified rhinitis. Therefore, the present study used the Chinese version of the 31-item Rhinosinusitis Outcome Measure (CRSOM-31) to investigate the disease-specific quality of life among patients using various CAM modalities for the management of their rhinitis.

## 2. Materials and Methods

### 2.1. Study Design and Subjects

This was a cross-sectional survey conducted from July to September 2011 at a medical center in central Taiwan. Patients with physician-diagnosed rhinitis from the outpatient department of otolaryngology were invited to participate in the study. The diagnosis of rhinitis was based on patients' reports of typical nasal symptoms persisting for two weeks or more and rhinoscopy examination. Physical examinations with anterior rhinoscopy or nasal endoscopy were performed by two rhinologists (Rong-San Jiang and Kai-Li Liang). All enrolled patients revealed signs of nasal inflammation including mucosal edema, nasal polyp, polypoid swelling, discharge, or crust. Exclusion criteria included patients who were under 20 years old and those with the following disorders including sinonasal tumors, asthma, chronic obstructive pulmonary disease, and tuberculosis. Participants were informed of the study protocol and allowed to be included in this study after their written consent. This study was approved by the Institutional Review Board of the Taichung Veterans General Hospital, Taiwan (no. CE1101).

### 2.2. Data Collection

Eligible patients were asked to complete a questionnaire with questions on demographic and lifestyle characteristics, disease-specific quality of life, and previous use of treatment modalities for rhinitis. The treatment modalities included conventional allopathic medicine and 17 CAM modalities. The disease-specific quality of life was assessed using the Chinese version of the Rhinosinusitis Outcome Measures (CRSOM-31) [17]. The CRSOM-31 is a validated instrument translated from the widely used Rhinosinusitis Outcome Measures 31 (RSOM-31), which contains seven domains including nasal symptoms, eye symptoms, sleep, ear symptoms, general symptoms, practical problems, and emotional consequences. Patients were asked to score each of the 31 items for its severity and importance to them, over the past two weeks. The total CRSOM-31 score was calculated as the sum of the severity multiplied by the importance scores. Higher scores indicate a greater impact of rhinosinusitis on quality of life [18].

### 2.3. Statistical Analysis

Continuous data were expressed as mean and standard deviation (SD) and categorical data were expressed as frequencies and percentages. The differences between the mean of continuous variables and distribution of categorical variables were evaluated using *t*-test and Pearson's chi-square test, respectively. The association of disease-specific quality of life and CAM use in patients with rhinitis was assessed by multivariate logistic regression analysis, adjusting for other potential confounders including demographic and lifestyle characteristics.

To reduce the number of intercorrelated variables of CAM modalities, factor analysis was conducted to identify the underlying patterns of CAM use. Factor analysis was performed using principal components extraction on treatment modalities (17 CAM modalities and conventional allopathic medicine) and with Varimax rotation and criteria including an eigenvalue >1, scree slope, and interpretability. The resulting factors were then evaluated in subsequent simple and multiple linear regression analyses with backward stepwise selection method. Other independent factors under evaluation included sex, age group, body mass index, marital status, educational level, alcohol use, smoking, regular exercise, and etiology of rhinitis. All computations were performed using SPSS version 21.0 for Windows (SPSS, Inc., Chicago, IL, USA). Two-tailed *P* values < 0.05 were considered statistically significant.

## 3. Results

### 3.1. Participant Characteristics and Factors Associated with CAM Modalities

A total of 288 patients with acute or chronic rhinosinusitis and allergic or nonallergic rhinitis consecutively attending the clinic were invited to participate the study and 279 (96.9%) were successfully interviewed. Of the 279 patients, 245 (87.8%) provided complete information.  Table 1 shows the basic characteristics, lifestyle factors, CRSOM-31 overall scores, and etiologies of rhinitis of the study sample. The mean age was 49.0 years and 59.2% were males. Significantly greater CRSOM-31 overall score, eye symptoms score, sleep score, general symptoms score, practical problems score, and emotional consequence score were observed in the female group whereas nasal symptoms score and ear symptoms score were not significantly different between males and females.

Table 2 presents the results of the factor analysis of the 17 CAM modalities and conventional allopathic medicine. Barlett's test of sphericity was significant (*P* < 0.001) and Kaiser-Meyer-Olkin (KMO) measure of sampling adequate was 0.75, indicating that the sample met the criteria for factor analysis. The factor analysis yielded a six-factor structure that explained 59.0% of the variance in the 18 items. Based on the modalities that were included in each of the factors, the six factors were labeled as (1) traditional Chinese medicine, (2) mind-body, (3) manipulative-based, (4) supernatural healings and phototherapy, (5) energy and diet, and (6) western medicine.

### 3.2. Changes in CRSOM-31 Overall Scores

The associations between CRSOM-31 scores and the six factors are shown in Table 3. Results of the simple linear regression analysis indicated that Factor 1, Factor 2, Factor 6, age, and sex were significantly associated with CRSOM-31 scores. Furthermore, multiple linear regression analysis found that Factor 1, Factor 2, Factor 3, female sex, and smoking were independent predictors of CRSOM-31 scores.

## 4. Discussion

In this cross-sectional study, we used the CRSOM-31 overall score to assess the predictors of disease-specific quality of life in patients with rhinitis. Key results indicated that female sex, smoking, and the use of different CAM modalities were independent predictors of worse quality of life. To the best of our knowledge, this is the first study to report the significant association between the disease-specific quality of life and use of different CAM modalities for the patients with rhinitis.

To facilitate the interpretation of CAM use, instead of evaluating the 17 CAM modalities and the conventional medicine as 18 different variables, we conducted a factor analysis to examine whether these modalities occurred in clusters. The analysis revealed six factors, which we have labeled as (1) traditional Chinese medicine, (2) mind-body, (3) manipulative-based, (4) supernatural healings and phototherapy, (5) energy and diet, and (6) western medicine (Table 2).

Female patients were found to experience a significantly greater impairment of the disease-specific quality of life compared with male patients. This finding is in consistent with previous research on health-related quality of life [19, 20]. When examining the scores of the seven CRSOM-31 domains between males and females, all of them except nasal and ear symptoms were significantly worse in female patients. One explanation could be that women have different illness perceptions and response to the disease [21].

Smoking was significantly associated a worse disease-specific quality of life in the present study. Previous large-scale population studies have shown that cigarette smoking and chronic rhinitis were associated in a dose-dependent manner [22]. Smoking should lead to increased symptoms therefore resulting in a worse disease-specific quality of life. A Swedish multicenter survey in patients with asthma found that current smoking is one of the significant independent factors associated with a worse quality of life [23]. Another study in Singapore also reported that adult patients with asthma who smoke were more affected by nocturnal symptoms, with lower emotional function scores in the Asthma Quality of Life (AQLQ) questionnaires [24]. A recent study on patients with chronic rhinosinusitis indicated that the proinflammatory effect of smoking could affect levels of inflammatory biomarkers systemically, in addition to locally on sinus mucosa [25]. Since these changes are potentially reversible, smoking cessation is strongly advised to improve the disease-specific quality of life in patients with rhinitis.

Of the six factors emerged from the factor analysis, Factor 1 (traditional Chinese medicine), Factor 2 (mind-body), and Factor 3 (manipulative-based) were significantly associated with a worse disease-specific quality of life, as indicated by a higher CRSOM-31 scores. Conversely, the other three factors, namely, supernatural healings and phototherapy, energy and diet, and western medicine, were not associated with disease-specific quality of life. The modalities included in Factor 1 appeared to deal with not only the disease treatment aspects of traditional Chinese medicine and acupuncture but also the traditional concept of maintaining body homeostasis in Chinese medicine [26]. The overall tonic care is thought to be crucial in treatment of disease and therefore it is not surprising to observe that the use of health food supplements including probiotics was included together with traditional Chinese medicine, acupuncture, and Chinese topical prescription within the same factor. Traditional Chinese medicine users are likely to value the importance of improving the overall health status through the use of health food supplements. In fact, health food supplements are often promoted along with preparation of Chinese herbal extracts for both health maintenance and disease treatment in the Chinese societies.

Factor 2 included modalities that are mind-body related. Previous research reported that among the patients attending a general otolaryngology clinic in the United Kingdom, the top-ranking nonherbal CAM modalities used in the preceding 12 months were massage (28%), followed by acupuncture (22%), and aromatherapy (16%) [27]. In another study of 288 Canadian patients with chronic rhinosinusitis, massage (33%) and aromatherapy (16%) ranked the third and fifth places in the use of CAM as a treatment for symptoms of chronic rhinosinusitis [28]. Therefore, the use of aromatherapy is relatively common. A study of 3,000 otolaryngology patients reported that 1.7% of them had experienced some form of complication induced by the use of essential oils [29]. Uses should be informed of the possible adverse effects of essential oils, particularly with its prolonged use.

Factor 2 also included folk remedy. Information of various Chinese folk remedies is widely available on the Internet. Not only that these claims have not been subjected to evaluations of their effectiveness, but also many may lead to serious adverse effect. For example, some remedies work on the principles of ablation of the inferior nasal concha to open up the blocked airways using corrosive preparation such as burnt alum and venenum bufonis. Nevertheless, uninformed patients who try to avoid surgery may try these remedies as they appear to offer temporarily symptom reduction. Health care workers should emphasis to their patients the availability of various treatment options in addition to surgical intervention.

Besides aromatherapy and folk remedy, Buddhist chanting or bible reading and prayer or meditation also emerged under Factor 2. Spirituality is a vital aspect of wellness, providing a sense of meaning in life, particularly during times of illness. Studies on patients with serious illness such as advanced chronic kidney disease [30] and cancer [31] have shown a positive association between spirituality and health-related quality of life. However, the results from a large prospective study among older adults representative of the general population found that prayer was not predictive of health-related quality of life [32]. In this study, a worse disease-specific quality of life was significantly associated with Factor 2, which included Buddhist chanting or bible reading and prayer or meditation in the factor. Whether the association is the results of the need to use spiritual practice to feel in control of the disease will require further investigations.

Factor 3 included three modalities that are manipulative based and they included Tui-na, Gua-sha or cupping therapy, and massage. The use of these modalities by patients with rhinitis could be associated with the belief that rhinitis is a result of qi stagnation and blood stasis. According to the traditional Chinese medicine theory, disorders in the respiratory system are often related to a deficiency of the lung, qi stagnation, and blood stasis [33]. Tui-na, Gua-sha or cupping therapy and massage are believed to be able to improve qi and thereby treating the disease. Therefore, patients with a worse disease-specific quality of life might use these manipulative-based modalities as a way to tonify their qi.

The strength of this study is the clinically verified rhinitis diagnosis and the validated quality of life instrument, but the results are also subject to a few limitations. First, the prevalence of CAM use might have been slightly underestimated since our sample was recruited from a hospital and therefore would exclude those individuals who had entirely avoided the use of conventional western medicine. Second, the possibility of a recall bias could not be ruled out with the use of self-report data. Third, since the study hospital was a medical center, it is possible that our sample represent cases of greater disease severity.

## 5. Conclusion

This study identified factors associated with a worse disease-specific quality of life in patients with clinically verified rhinitis. In addition to female sex and smoking, three factors obtained from the factor analysis of 17 CAM modalities were found to be associated with a lower disease-specific quality of life. Findings from this study can help health care providers to understand CAM use in their patients in relation to their disease-specific quality of life and therefore be able to provide advices in the choice and safety of CAM use for their patients.

## Figures and Tables

**Table 1:** Characteristics of study participants (*N* = 245).

Variable	*n* (%)			*P*
	Total	Male	Female	
		145 (59.2%)	100 (40.8%)	
Age (year), mean (SD)	49.0 (18.4)	49.7 (19.6)	47.9 (16.4)	0.451
[median, min–max]	[49.0, 20–89]	[47.0, 20–89]	[51.0, 20–85]	
Age category (year)				0.064
20–30	46 (18.8)	28 (19.3)	18 (18.0)	
31–40	40 (16.3)	24 (16.6)	16 (16.0)	
41–50	43 (17.6)	29 (20.0)	14 (14.0)	
51–60	58 (23.7)	25 (17.2)	33 (33.0)	
61–89	58 (23.7)	39 (26.9)	19 (19.0)	
Body mass index (kg/m^2^), mean (SD)	24.0 (3.8)	24.8 (3.5)	22.8 (3.9)	<0.001
[median, min–max]	[23.8, 12.3–35.6]	[24.2, 17.2–35.0]	[22.7, 12.3–35.6]	
Body mass index category				0.005
Underweight	11 (4.5)	3 (2.1)	8 (8.0)	
Normal	126 (51.4)	66 (45.5)	60 (60.0)	
Overweight	59 (24.1)	41 (28.3)	18 (18.0)	
Obese	49 (20.0)	35 (24.1)	14 (14.0)	
Marital status				0.488
Single, divorced, widowed, or other	79 (32.2)	44 (30.3)	35 (35.0)	
Married	166 (67.8)	101 (69.7)	65 (65.0)	
Educational level				0.147
Elementary school or below	68 (27.8)	35 (24.1)	33 (33.0)	
High school or above	177 (72.2)	110 (75.9)	67 (67.0)	
Smoking				<0.001
No	203 (82.9)	109 (75.2)	94 (94.0)	
Yes	42 (17.1)	36 (24.8)	6 (6.0)	
Alcohol use				<0.001
No	182 (74.3)	88 (60.7)	94 (94.0)	
Yes	63 (25.7)	57 (39.3)	6 (6.0)	
Exercise regularly				0.305
No	43 (17.6)	22 (15.2)	21 (21.0)	
Yes	202 (82.4)	123 (84.8)	79 (79.0)	
CRSOM-31 overall score, mean (SD)	168.4 (100.0)	153.0 (97.8)	190.8 (99.4)	0.003
[median, min–max]	[150.0, 16–472]	[123.0, 16–428]	[178.0, 45–472]	
Nasal symptoms score, mean (SD)	40.9 (23.6)	39.8 (24.7)	42.7 (21.9)	0.342
[median, min–max]	[38.0, 0–108]	[34.0, 0–108]	[41.5, 2–104]	
Eye symptoms score, mean (SD)	8.4 (9.6)	6.8 (10.8)	8.3 (11.0)	0.002
[median, min–max]	[4.0, 0–40]	[4.0, 0–32]	[8.0, 0–40]	
Sleep score, mean (SD)	20.9 (17.8)	18.2 (17.7)	24.8 (17.4)	0.004
[median, min–max]	[18.0, 0–60]	[13.0, 0–60]	[24.0, 0–60]	
Ear symptoms score, mean (SD)	15.5 (16.0)	14.4 (15.6)	17.2 (16.6)	0.178
[median, min–max]	[11.0, 0–66]	[9.0, 0–66]	[12.0, 0–65]	
General symptoms score, mean (SD)	33.8 (28.2)	29.1 (25.1)	40.7 (31.0)	0.002
[median, min–max]	[28.0, 0–135]	[24.0, 0–110]	[34.0, 0–135]	
Practical problems score, mean (SD)	27.9 (19.8)	25.8 (20.1)	31.0 (19.1)	0.044
[median, min–max]	[24.0, 0–91]	[23.0, 0–91]	[27.0, 0–80]	
Emotional consequences score, mean (SD)	20.9 (17.8)	19.0 (17.6)	23.6 (17.7)	0.046
[median, min–max]	[19.0, 0–60]	[12.0, 0–60]	[24.0, 0–60]	
Etiology of rhinitis				
Allergic rhinitis				0.966
No	118 (48.2)	70 (48.3)	48 (48.0)	
Yes	127 (51.8)	75 (51.7)	52 (52.0)	
Deviation of nasal septum				0.007
No	204 (83.3)	113 (77.9)	91 (91.0)	
Yes	41 (16.7)	32 (22.1)	9 (9.0)	
Chronic hypertrophic rhinitis				0.129
No	168 (68.6)	94 (64.8)	74 (74.0)	
Yes	77 (31.4)	51 (35.2)	26 (26.0)	
Chronic rhinosinusitis				0.299
No	113 (46.1)	71 (49.0)	42 (42.0)	
Yes	132 (53.9)	74 (51.0)	58 (58.0)	

Values are frequencies (percentages), unless otherwise specified.

SD: standard deviation; min: minimum; max: maximum.

**Table 2:** Seven-factor solution for the 17 complementary medicine modalities and conventional allopathic medicine use.

Factor	% variance explained	Item	Factor loading					
			1	2	3	4	5	6
(1) Traditional Chinese medicine	11.7	Acupuncture and moxibustion	0.783					
		Traditional Chinese medicine	0.765					
		Chinese topical prescription Sang-fu-teh	0.617					
		Health food supplements including probiotics	0.502					

(2) Mind-body	11.4	Buddhist chanting or bible reading		0.638				
		Prayer or meditation		0.632				
		Aromatherapy		0.620				
		Folk remedy		0.558				

(3) Manipulative-based	11.1	Tui-na			0.834			
		Gua-sha (scraping) or cupping therapy			0.798			
		Massage			0.708			

(4) Supernatural healings and phototherapy	9.8	Tai-sui pacifying ritual				0.792		
		Phototherapy				0.695		
		Shou-jing ritual				0.599		

(5) Energy and diet	9.2	Yoga, Tai chi, and Qigong					0.814	
		Music therapy					0.603	
		Special diet					0.390	

(6) Western medicine	6.0	Conventional allopathic medicine						0.864

**Table 3:** Simple linear regression and multiple linear regression analyses of CRSOM-31 score (per 10 units).

Variable	Simple linear regression analysis		Multiple linear regression analysis	
	*β* (95% CI)	*P*	*β* (95% CI)	P
Factor 1	2.05 (0.82, 3.29)	0.001	1.93 (0.74, 3.11)	0.002
Factor 2	1.80 (0.56, 3.04)	0.005	1.58 (0.39, 2.76)	0.009
Factor 3	1.07 (−0.19, 2.32)	0.096	1.37 (0.18, 2.56)	0.024
Factor 4	0.39 (−0.88, 1.65)	0.546		
Factor 5	−0.24 (−1.51, 1.02)	0.705		
Factor 6	1.26 (0.01, 2.52)	0.049		
Age (/10 year)	−0.95 (−1.63, −0.27)	0.006		
Sex (females versus males)	3.78 (1.26, 6.30)	0.003	4.54 (2.05, 7.04)	<0.001
Body mass index (kg/m^2^)	−0.22 (−0.55, 0.11)	0.194		
Marital status^*^	1.95 (−0.74, 4.44)	0.154		
Educational level^**^	1.53 (−1.28, 4.34)	0.285		
Smoking	3.10 (−0.23, 6.42)	0.068	4.37 (1.12, 7.62)	0.009
Alcohol use	−0.58 (−3.47, 2.30)	0.692		
Exercise regularly	−0.54 (−3.86, 2.77)	0.747		
Etiology of rhinitis				
Allergic rhinitis	1.24 (−1.28, 3.76)	0.332		
Deviation of nasal septum	−0.60 (−3.97, 2.78)	0.729		
Chronic hypertrophic rhinitis	−0.96 (−3.68, 1.75)	0.486		
Chronic rhinosinusitis	2.48 (−0.03, 4.99)	0.053		

The variable “general health status" was not included in the model development because of its strong correlation with the CRSOM-31 scores.

^*^Single, divorced, widowed, or other versus married.

^**^Elementary school or below versus high school or above.
